# Nematode Functional Genomics

**DOI:** 10.1002/(SICI)1097-0061(200004)17:1<43::AID-YEA1>3.0.CO;2-Z

**Published:** 2000

## Abstract

*Alan Coulson* has two main roles at the Sanger Centre, revolving around the worm and the human genome projects. Although the worm sequence is essentially finished, the tidying-up of that and the physical map is ongoing. There is also a continuous need for communication with the worm field with regard to information and materials relating to the sequence project. For example, the cosmids and YACs of the physical map continue to be, as they have been for many years now, an extremely powerful resource, and the Sanger Centre distributes in the order of 500 clones per month to the community.

Alan is team leader of the worm functional genomics group, which is currently small but will be expanding shortly. Patricia Kuwabara is a member of the team and a description of their activities can be found below. The Human Genome Project is sequencing mapped PAC and BAC clones. Alan's primary involvement is with the team that is responsible for subcloning the 10 000 or so clones that will be required to complete the one-third of the genome sequence to be contributed by the Sanger Centre.

*Patricia Kuwabara* has been using * Caenorhabditis elegans* as a model for understanding how protein–protein interactions regulate cell-to-cell signalling. Her research has focused on understanding the molecular mechanisms underlying the genetics of *C. elegans* sex determination. This work has led into a study of regulated proteolysis involving calpains and also into the roles of the multiple *C. elegans* Patched proteins, which in other organisms have been shown to be receptors for the Hedgehog morphogen.

In addition, the group is taking advantage of the completion of the *C. elegans* genome sequence to develop whole genome DNA microarrays for expression profiling. At the Sanger Centre, DNA microarrays are providing opportunities to examine how development and physiology are regulated globally, because most nematode genes have now been identified at the sequence level. The group are being assisted in this endeavour by Dr Stuart Kim (Stanford, CA).


**Yeast**
*Yeast* 2000; **17**: 43–47.

The Sanger Centre

Interview: Alan Coulson and Patricia Kuwabara

### Nematode functional genomics

Alan Coulson **has two main roles at the Sanger Centre, revolving around the worm and the human genome projects. Although the worm sequence is essentially finished, the tidying-up of that and the physical map is ongoing. There is also a continuous need for communication with the worm field with regard to information and materials relating to the sequence project. For example, the cosmids and YACs of the physical map continue to be, as they have been for many years now, an extremely powerful resource, and the Sanger Centre distributes in the order of 500 clones per month to the community.**

**Alan is team leader of the worm functional genomics group, which is currently small but will be expanding shortly. Patricia Kuwabara is a member of the team and a description of their activities can be found below. The Human Genome Project is sequencing mapped PAC and BAC clones. Alan's primary involvement is with the team that is responsible for subcloning the 10 000 or so clones that will be required to complete the one-third of the genome sequence to be contributed by the Sanger Centre.**

Patricia Kuwabara **has been using *Caenorhabditis elegans* as a model for understanding how protein–protein interactions regulate cell-to-cell signalling. Her research has focused on understanding the molecular mechanisms underlying the genetics of *C. elegans* sex determination. This work has led into a study of regulated proteolysis involving calpains and also into the roles of the multiple *C. elegans* Patched proteins, which in other organisms have been shown to be receptors for the Hedgehog morphogen.**

**In addition, the group is taking advantage of the completion of the *C. elegans* genome sequence to develop whole genome DNA microarrays for expression profiling. At the Sanger Centre, DNA microarrays are providing opportunities to examine how development and physiology are regulated globally, because most nematode genes have now been identified at the sequence level. The group are being assisted in this endeavour by Dr Stuart Kim (Stanford, CA).**

***CFG: Can you comment on the relative efficacy of the various alternative mutagenesis methods available for studying* C. elegans*, for example Tc1 insertion and RNA interference techniques? What prompted you to choose the random mutagenesis method you are using?***

AC/PK: A complete set of strains carrying knockouts of every gene is frequently given as *sine qua non* for the post-genomics of any model organism. In the worm, it has not yet been possible to produce knockouts using transgenesis and homologous recombination, as is, of course, the case for yeast. Consequently, the emphasis is to develop high-throughput PCR-based methods that depend upon selection of the target mutation from a library of mutated animals. The ability to freeze and recover worms is obviously important in designing possible large-scale strategies. Although the imprecise excision of Tc1 transposons from the genome has for some time been a widely used method for the isolation of deletion mutants, there are drawbacks to the use of this approach on a large scale in that it is a two-step process—the isolation of the insertion allele followed by detection amongst the progeny of an appropriate excision event. This is a difficult process to scale-up.

More recently, the emphasis has been on the one-step process of detection by PCR of the targeted deletion in a library of chemically mutagenized animals. These methods were developed in the laboratories of Ronald Plasterk, Bob Barstead and Carl Johnson (Jansen *et al*., 1997; Liu *et al*., 1999). Currently, the most popular mutagen is trimethyl-

Copyright © 2000 John Wiley & Sons, Ltd.

psoralen and exposure to UV light. Deletions in the desired size-range of 500 bp–3 kb are found at a frequency of perhaps 1 in half a million genomes (which efforts are being made to improve). In order to screen the necessary number of genomes in a manageable way, microtitre plate cultures derived from a number of mutagenized worms (say 10 or 20) are grown. A part of the culture is frozen and the remainder are pooled in a multidimensional manner (there is a variety of schemes) for DNA extraction and PCR analysis. Following detection of a candidate deletion, the worms from the relevant well are thawed and sib-selected by further PCR.

These and related methods are certainly applicable by individual labs—a number of targets can be addressed rapidly in 'living' libraries without recourse to a frozen bank, for example—but a number of labs, including the Sanger Centre, have formed a consortium to pilot a project with the aim of producing large numbers of mutant strains. Although a knockout of every gene might be desirable, some knockouts are inevitably felt to be more desirable than others and the consortium is initially targeting genes by request. The need for this resource is clear from the large number of requests that greatly outstrips our current ability to supply. All the consortium labs are as yet optimizing protocols and methodologies, in particular to improve the frequency of appropriate deletions. Ideally, the strains derived by this project would be back-crossed and balanced. Currently, this is left to the recipient, as is, consequently, the phenotypic analysis. Information about the consortium can be found at **http://www.cigenomics.bc.ca/elegans/**

One of the most exciting developments in *C. elegans* has been the advent of RNA interference, both from the point of view of its utility and the exploration of the mechanisms involved (Fire *et al.*, 1998). The ability to rapidly phenocopy a null mutation, albeit transiently, in the vast majority of genes (some genes expressed in the nervous system, for example, appear to be refractory) is having a major impact on genomic analysis in the worm. While gonadal injection is the most efficient route, RNAi functions in worms that are soaked in the dsRNA (Tabara *et al.*, 1998) and by feeding worms with *Escherichia coli* that are expressing the dsRNA (Timmons and Fire, 1998). This latter may have application in large-scale drug screens. In order to circumvent the non-heritability of RNAi effects, and to broaden potential applications, Tavernarakis *et al.* (2000) have employed transgenic inverted copies of gene sequence from which RNA expression was driven by heat-shock induction, giving rise to an RNAi-effective hairpin RNA.

The relative ease of the methodologies has led to schemes aimed at the RNAi screening of all 19 000 genes. At the Sanger Centre (with MRC funding) we have a collaboration with the Hyman lab at EMBL to identify, primarily, genes involved in cell division. For completeness (only 50% of predicted *C. elegans* genes have an identified cDNA), promoter-tailed PCR products are made from genomic DNA template and double-stranded RNA is derived from these fragments. In the Hyman lab, detailed analysis of the first two cell divisions of the embryos from RNA-injected mothers is enabled by time-lapse video microscopy, and a less detailed analysis of later phenotypes is also made. To date, 95% of the 2335 predicted genes on chromosome III have been analysed. Preliminary results show that over 300 (14%) genes gave rise to a phenotype, of which approximately half were recorded as aberrations in the first two cell divisions. The intention is to continue this analysis across the entire genome.

***CFG: What proportion of essential genes and genes with no phenotype have been seen in* C. elegans *gene deletion studies so far? Do you expect these numbers to improve as a result of your large-scale project?***

***Yeast has proved to have many genes with no apparent phenotype. This is possibly due to high levels of redundancy, or to genes having very specific expression. Do you think either of these will apply to* C. elegans*?***

AC/PK: Around 50% of genes show no obvious phenotype when knocked out. But to some extent this number obviously depends upon how hard you look and what you look for—how large a variety of hoops you put the worms through. There is a rich variety of mutant phenotypes in *C. elegans* that can be scored with the aid of a microscope and the trained eye of the investigator. The nature of these phenotypes often provide clues to help researchers determine the normal wild-type function of a gene. The three-letter gene name assigned to a mutant has historically described the loss-of-function or absence-of-function phenotype, although since the completion of the *C. elegans* genome sequence, many predicted genes are now named because they

Copyright © 2000 John Wiley & Sons, Ltd. 

share sequence similarity with a previously studied gene.

Many of the more easily recognized phenotypes are associated with morphological mutants that display abnormalities in movement, pattern formation, size, or shape. However, the worm also displays chemotaxis and other behavioural phenotypes that are also capable of being scored. Mutations in essential genes usually cause embryonic or larval lethality; however, sterile adults unable to reproduce would also be characterized as lethal. To pursue a more detailed analysis of a mutant phenotype, there are a variety of tissue-specific expression reporters (LacZ and GFP) and antibodies that are available to help investigators determine how a specific mutation affects gene expression and pattern formation. Video and 4-D microscopic techniques are also being used to record and analyse lineage and behavioural defects. The development of new worm sorting and recording tools raises the exciting possibility that future phenotypic analyses will be automated and provide more opportunities for researchers to detect subtle phenotypes. Combinations of knockouts can be used to address the effects of redundancy.

***CFG: What are your views on the relative value of applying other functional genomics approaches to* C. elegans*, such as complementation studies, microarray technology and expression pattern studies? Do you see them as complementary to your project?***

At present, large-scale complementation studies in *C. elegans* are not yet feasible. However, complementation studies have been performed successfully on a gene-by-gene basis. For example, it has been shown by the Greenwald lab (Columbia University, NY) that the human presenilin gene (PS1 or PS2), which is associated with early-onset Alzheimer's disease, can effectively replace the activity of the *C. elegans* orthologue sel-12 (for suppressor and/or enhancer of lin-12) (Levitan *et al.*, 1996). In terms of other global applications, microarrays are being used successfully for expression profiling and yeast two-hybrid screens for identifying protein–protein interactions are being attempted on a genome-wide scale in the lab of Marc Vidal (Harvard, MA) (Walhout *et al.*, 2000). GFP and LacZ reporters are being used to analyse the expression patterns of specific genes. Other methods, such as *in situ* hybridization, RT–PCR, and protein immunolocalization with antibodies, are also widely used for gene characterization. These techniques are complementary in the sense that they add to our understanding about the temporal and spatial requirements of individual genes, but they do not replace the need for deletion knockouts or loss-of-function mutations, which tell us about the function of a gene and whether it plays an essential role in development.

The *C. elegans* whole genome microarray project at the Sanger Centre is being funded by the Biotechnology and Biological Sciences Research Council (BBSRC). Our intention is to provide the microarray technology as a resource to the *C. elegans* research community so that the expense of the technique, which is perceived by many to be relatively high, would not be a deterrent to its use. The *C. elegans* microarray will consist of exon-rich PCR-amplified genomic products representing all predicted ORFs and will be generated essentially by following protocols developed in the lab of Pat Brown (Stanford). We are still in the early days of the project and are busy optimizing various steps in the procedure, particularly RNA sample preparation. Unlike yeast, *C. elegans* is a multicellular organism that advances though a number of life stages, therefore proportionately more attention must be devoted to sample preparation in order to generate meaningful and reproducible results. However, preliminary reports from the lab of Stuart Kim (Stanford, CA) indicate that microarrays clearly deserve a place in the repertoire of functional tools for analysing the genome of *C. elegans*. Therefore, we are optimistic that the *C. elegans* microarrays will allow researchers to take advantage of the completed genome sequence and to investigate how development and physiology are regulated globally.

***CFG: Do you think that a combination of such approaches will be the best way forward, or do you think that some of them will need to be improved or replaced?***

AC/PK: It is axiomatic to say that a combination of approaches will be the best way forward because it provides opportunities to learn about gene activity and regulation from different perspectives. It never hurts to have corroborating data, if in some cases the information output is overlapping. It is also the case that not all methods have been adapted for

Copyright © 2000 John Wiley & Sons, Ltd. 

high throughput analysis. Therefore, it is still the expectation that individual labs will continue to play a major role in providing a detailed analysis of selected genes.

Obviously, we benefit from methods that are more efficient because they are faster, more sensitive, more reliable, less technically demanding and even less expensive; in most cases, the evolution of new techniques is driven by the needs of the investigator. For example, RNAi is one of the most powerful tools available for obtaining rapid insights into gene function in *C. elegans*. At the same time, some laboratories have been unable to take advantage of this method because they lack the facilities or the technical skill to perform microinjection. The Fire and Mello lab have recently shown that it is possible to elicit RNAi responses by soaking or feeding worms bacteria producing dsRNA (Tabara *et al.*, 1998; Timmons and Fire, 1998). These modifications will probably have the effect of enabling investigators to develop high throughput methods for performing RNAi, and also of increasing the accessibility of the technique to other labs.

On the other hand, old techniques should not necessarily be replaced by the new. Historically, *C. elegans* has attracted many investigators because of its genetic tractability. Oftentimes, partial loss-of-function and rare gain-of-function mutations have provided novel insights into gene function and regulation that could not be obtained by examining mutations that completely abolish gene activity. Moreover, powerful suppressor and enhancer screens for genetic modifiers, which have led to the identification of interacting genes in regulatory pathways, also rely on the ability to ameliorate or enhance the phenotypes of weak partial loss-of-function mutants. Therefore, although the ability to rapidly generate deletion mutants is an important aspect of whole genome analysis, a more detailed understanding of gene activity may still require a return to basics. It is also the case that genetic studies using *C. elegans* would benefit from the development of homologous gene replacement techniques.

***CFG: How useful would you say* C. elegans *has been so far in the study of more complex organisms?***

AC/PK: *C. elegans* has played an important role in the study of more complex organisms on a number of levels. First, the *C. elegans* genome sequencing project has provided a test bed for developing the various genome mapping, DNA sequencing and bioinformatic technologies that are now the foundation of high throughput genome sequencing projects, such as the human genome sequencing project. Second, many of the same genes and regulatory pathways found in more complex organisms are also present in *C. elegans*. However, unlike humans, the genetic tractability of *C. elegans* has meant that it is possible to order the genes involved in these pathways into regulatory hierarchies and also to identify additional regulators or modifiers through genetic screens and selections. *C. elegans* has made significant contributions to the understanding of cell signalling, programmed cell death, ageing and caloric restriction, neuronal wiring and synaptic properties, neurodegeneration and muscle biology.

Finally, it has been possible to replace the activity of a *C. elegans* gene with its human orthologue. This has allowed investigators to study specifically the human gene and also to show that the activity and regulation of the orthologue have been conserved. The ability to perform gene substitutions also opens the door to using the worm for drug screening.

***CFG: What can the study of the development of* C. elegans*, which has a rigidly defined cell lineage, tell us about the development of more complex organisms?***

AC/PK: Although *C. elegans* undergoes highly regulated development, lineage studies and cell killing by laser ablation have shown the importance of cell–cell interactions in regulating cell fate decisions and they have also revealed that the fate of some cells is to undergo a genetic programme of cell death. Subsequent studies of these processes have revealed that many of the pathways and molecules found in *C. elegans* also play similar roles in more complex organisms. Because the genome of *C. elegans* has undergone fewer wide-scale duplications, it is sometimes easier to demonstrate a correlation between a gene and a specific function. For example, it was possible to demonstrate the importance of caspases in programmed cell death because a single caspase, CED-3, is essential for this process in *C. elegans*, whereas there are multiple caspases in mammalian cells (Yuan *et al.*, 1993).

Copyright © 2000 John Wiley & Sons, Ltd.

***CFG: Do you envisage any further comparative genomics applications, such as the use of the genome sequence to identify new drug targets for human disease? How well does* C. elegans *work as a model organism; for example, do you think that methods based on complementation are likely to work?***

AC/PK: There are many areas where comparative genomics has the potential to facilitate medical intervention in human disease. First, although complementation studies on a large scale are not currently feasible, it is possible to replace a nematode gene with a human orthologue (e.g. presenilins). In situations where the human orthologue can replace the activity of the nematode gene, the nematode can now be used in screens to identify drugs that can suppress gene activity or ameliorate the activity of a partial loss-of-function mutation. It is also possible to use the nematode to identify mutations that confer resistance to the effects of a drug. This type of study can reveal important information about the mode of action of novel compounds.

Second, there are many filiarial diseases that affect humans. The availability of complete genome sequences for these parasites means that we will be able to identify metabolic pathways or gene families that may be unique to nematodes. Some of these genes may be excellent candidates for drug and vaccine intervention.

***CFG: Comparisons with parasitic nematodes have been suggested as way of identifying the genes responsible for the difference between these nematodes and the free-living* C. elegans. *Do you think that this approach will work?***

AC/PK: This approach has been used successfully for bacterial genomes and has led to the identification of pathogenicity islands, so it seems likely that this approach will also yield information about the differences between parasitic and free-living nematodes.

### References

Fire A, Xu SQ, Montgomery MK, Kostas SA, Driver SE, Mello CC. 1998. Potent and specific genetic interference by double-stranded RNA in *Caenorhabditis elegans*. *Nature* **391**: 806–811.

Jansen G, Hazendonk F, Thijssen KL, Plasterk RHA. 1997. Reverse genetics by chemical mutagenesis in *Caenorhabditis elegans*. *Nature Genet* **17**: 119–121.

Levitan D, Doyle TG, Brousseau D, *et al*. 1996. Assessment of normal and mutant human presenilin function in *Caenorhabditis elegans*. *Proc Natl Acad Sci USA* **93**: 14940–14944.

Liu LX, Spoerke JM, Mulligan EL, *et al*. 1999. High-throughput isolation of *Caenorhabditis elegans* deletion mutants. *Genome Res* **9**: 859–867.

Tabara H, Grisok A, Mello CC. 1998. RNAi in *C. elegans*: soaking in the genome sequence. *Science* **282**: 430–431.

Tavernarakis N, Wang SL, Dorovkov M, Ryazanov A, Driscoll M. 2000. Heritable and inducible genetic interference by double-stranded RNA encoded by transgenes. *Nat Genet* **24**: 180–183.

Timmons A, Fire AS. 1998. Specific interference by ingested dsRNA. *Nature* **395**: 854.

Walhout AJM, Sordella R, Lu X, *et al*. 2000. Protein interaction mapping in *C. elegans* using proteins involved in vulval development. *Science* **287**: 116–122.

Yuan J, Shaham S, Ledoux S, Ellis HM, Horvitz HR. 1993. The *C. elegans* cell death gene ced-3 encodes a protein similar to mammalian interleukin-1$\beta$-converting enzyme. *Cell* **75**: 641–652.

---

The interview articles of *Comparative and Functional Genomics* aim to present a commentary on topical issues in genomics studies. They are a personal critical analysis, by the interviewee, of current work in their field of expertise, and aim to provide implications for future genomics studies.

This interview was undertaken by Dr Joanne Wixon (Managing Editor). The Sanger Centre logo is reproduced here with the kind permission of The Sanger Centre.

Many thanks to:

**Alan Coulson, Patricia Kuwabara and John Sulston**
The Sanger Centre, Wellcome Trust Genome Campus, Hinxton, Cambridge CB10 1SA, UK.
**http://www.sanger.ac.uk/Projects/C_elegans**

Copyright © 2000 John Wiley & Sons, Ltd.
